# Cantrell Syndrome—A Rare Complex Congenital Anomaly: A Case Report and Literature Review

**DOI:** 10.3389/fped.2018.00201

**Published:** 2018-07-17

**Authors:** Claudiu Mărginean, Cristina Oana Mărginean, Liliana Gozar, Lorena Elena Meliţ, Horaţiu Suciu, Horea Gozar, Andrada Crişan, Manuela Cucerea

**Affiliations:** ^1^Department of Obstetrics and Gynecology, University of Medicine and Pharmacy Tîrgu Mureş, Tîrgu Mureş, Romania; ^2^Department of Pediatrics, University of Medicine and Pharmacy Tîrgu Mures, Tîrgu Mures, Romania; ^3^Department of Pediatric Cardiology, University of Medicine and Pharmacy Tîrgu Mures, Tîrgu Mures, Romania; ^4^Department of Cardiovascular Surgery, University of Medicine and Pharmacy Tîrgu Mures, Tîrgu Mures, Romania; ^5^Department of Pediatric Surgery, University of Medicine and Pharmacy Tîrgu Mures, Tîrgu Mures, Romania; ^6^Department of Neonatology, University of Medicine and Pharmacy Tîrgu Mureş, Tîrgu Mureş, Romania

**Keywords:** Cantrell syndrome, ectopia cordis, prenatal diagnosis, fetal screening, ultrasound examination

## Abstract

Cantrell syndrome (CS) or pentalogy of Cantrell is defined as a rare condition involving a midline anterior abdominal wall defect, a distal sternal cleft, a defect of the anterior diaphragm, and a defect of the apical pericardium with pericardio-peritoneal communication, as well as intracardiac anomalies. We report the case of a male newborn with type 2 CS diagnosed during intrauterine life based on ultrasonographic evaluation. Clinical examination at birth revealed an abdominal wall defect with extrathoracic displacement of the heart and a diastasis of the sagittal suture. Postnatal echocardiography revealed tricuspid atresia, partial extrathoracic and extra-abdominal displacement of the heart and liver, a large ventricular septal defect, severe subpulmonary stenosis, hypoplasia of the pulmonary artery, and a large hourglass-shaped left ventricle secondary to narrowing of the heart at the level of its extrathoracic displacement. Computed tomography showed additional abnormalities including increased left ventricular volume with extrathoracic apical aneurysmal dilatation below the xiphoid process at the level of anterior abdominal wall, a hypoplastic right ventricle, partial transparietal herniation of the left hepatic lobe adjacent to a left ventricular diverticulum, and an adrenal hematoma. The newborn received intensive medical management during his first week of life; however, surgical management had to be postponed owing to his unstable condition. Eventually, it was performed on the 14th day of life, but unfortunately, the newborn died shortly after the procedure.

## Introduction

Cantrell syndrome (CS) or pentalogy of Cantrell was first described in 1958. It involves multiple congenital anomalies such as a midline anterior abdominal wall defect, a distal sternal cleft, a defect in the anterior diaphragm, and a defect of the apical pericardium with pericardio-peritoneal communication, as well as intracardiac anomalies (1). The prevalence of this syndrome varies between 1:65,000 and 1:200,000 cases (2), although all patients do not present with all the aforementioned anomalies. Cantrell et al., when they first described the syndrome, attributed these anomalies to developmental failure between 14 and 18 days of embryonic life involving an inappropriate differentiation of a segment of the lateral mesoderm (1); however, the exact pathogenesis of this condition remains unclear. Nevertheless, this syndrome is considered to show a multifactorial etiology. In a few cases, ventral defects can be associated with craniofacial defects and the limb-body wall complex, a fatal condition, with a prevalence of only 0.2–3.3:10,000 live births (3). Recent studies have demonstrated a mutation in the *Porcupine* homolog gene in patients with CS and focal dermal hypoplasia, (also known as the Goltz-Gorlin syndrome) (4) and in those with the limb-body wall complex syndrome (5). *Porcupine*, an O-acyltransferase involved in the acylation of Wnt ligands, is located on the X chromosome, and its deletion in mice is known to cause focal dermal hypoplasia and developmental anomalies of the body wall (6); however, it is unclear if mutations in *Porcupine* also cause CS. A recent study has demonstrated the role of Wnt ligands in the pathogenesis of an unfused sternum and ectopia cordis (EC) (7), the 2 features that are defining components of CS.

CS was classified by Toyama into: class I–the occurrence of all 5 defects, class II–the occurrence of 4 defects with intracardiac and ventral abdominal wall abnormalities definitely present, and class III–incomplete expression of the disorder showing various combinations of defects, although sternal anomalies are definitely present (8).

EC, a typical feature of CS, is defined as a partial or complete displacement of the fetal heart outside the thoracic cavity. Studies report a prevalence of approximately 7.9:1 million live births (9). Depending upon the cardiac location, EC has been categorized into four types: cervical–the most uncommon (3% of all cases), thoracic–the most common (60%), abdominal (30%), and thoracoabdominal (7%) (10). This rare entity represents only 0.1% of all congenital heart anomalies (11).

Despite considerable advances in the management of CS, the survival rate is fairly low and it depends primarily upon the type and severity of associated anomalies.

We report a case of an incomplete CS and highlight the role/importance of prenatal diagnosis and the wide variety of anomalies associated with this syndrome.

*Informed consent was obtained from the patient's mother (legal guardian) for the publication of this case report*.

## Case report

### Presenting concerns

A 31-year-old woman, gravida 2 para 1, presented for a prenatal ultrasonographic examination at 36 gestational weeks owing to a suspicion of a fetal thoracic wall defect. Her personal history revealed a spontaneous abortion and no consanguinity. She underwent routine ultrasonographic examinations at 13, 22, and 30 gestational weeks at a regional hospital; however, at 35 gestational weeks, ultrasonography revealed an abnormal fetal thoracic wall.

**Figure 1:**
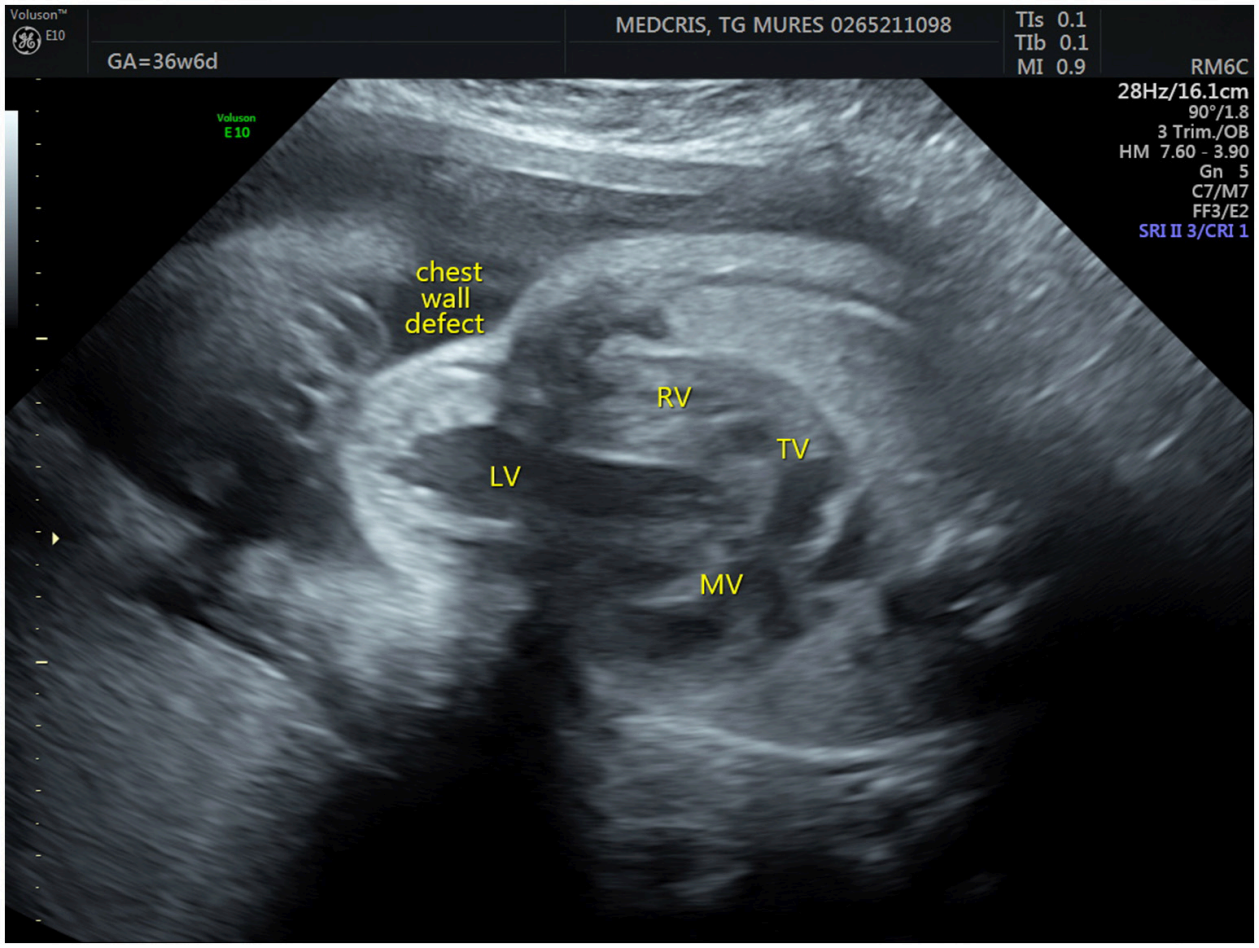
Fetal ultrasonographic image shows an extrathoracic left ventricle (RV, right ventricle; LV, left ventricle; MV, mitral valve; TV, tricuspid valve).

**Figure 2:**
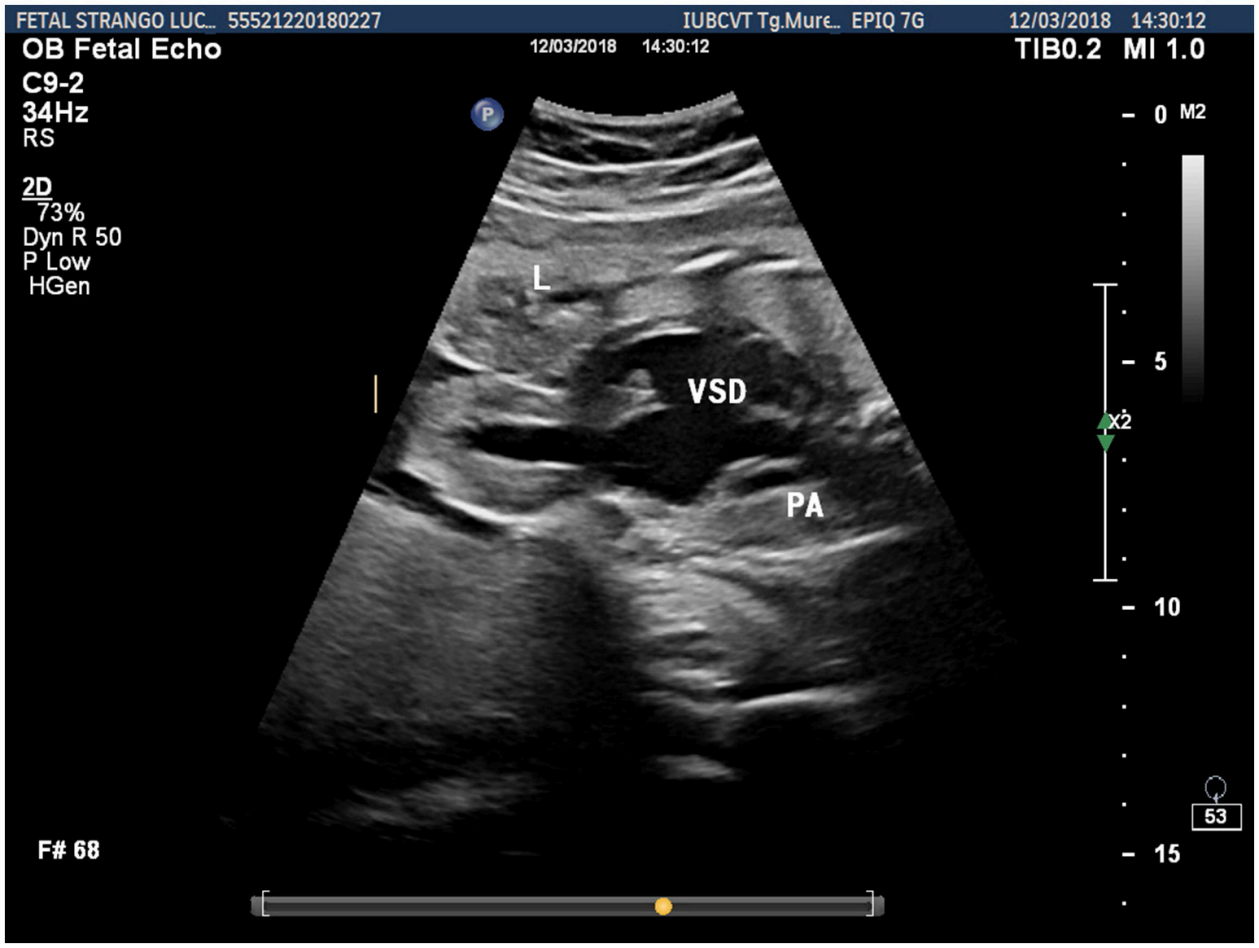
Fetal ultrasonographic image shows an extrathoracic left ventricle, a ventricular septal defect and pulmonary artery hypoplasia (L, liver; VSD, ventricular septal defect; PA, pulmonary artery).

**Figure 3:**
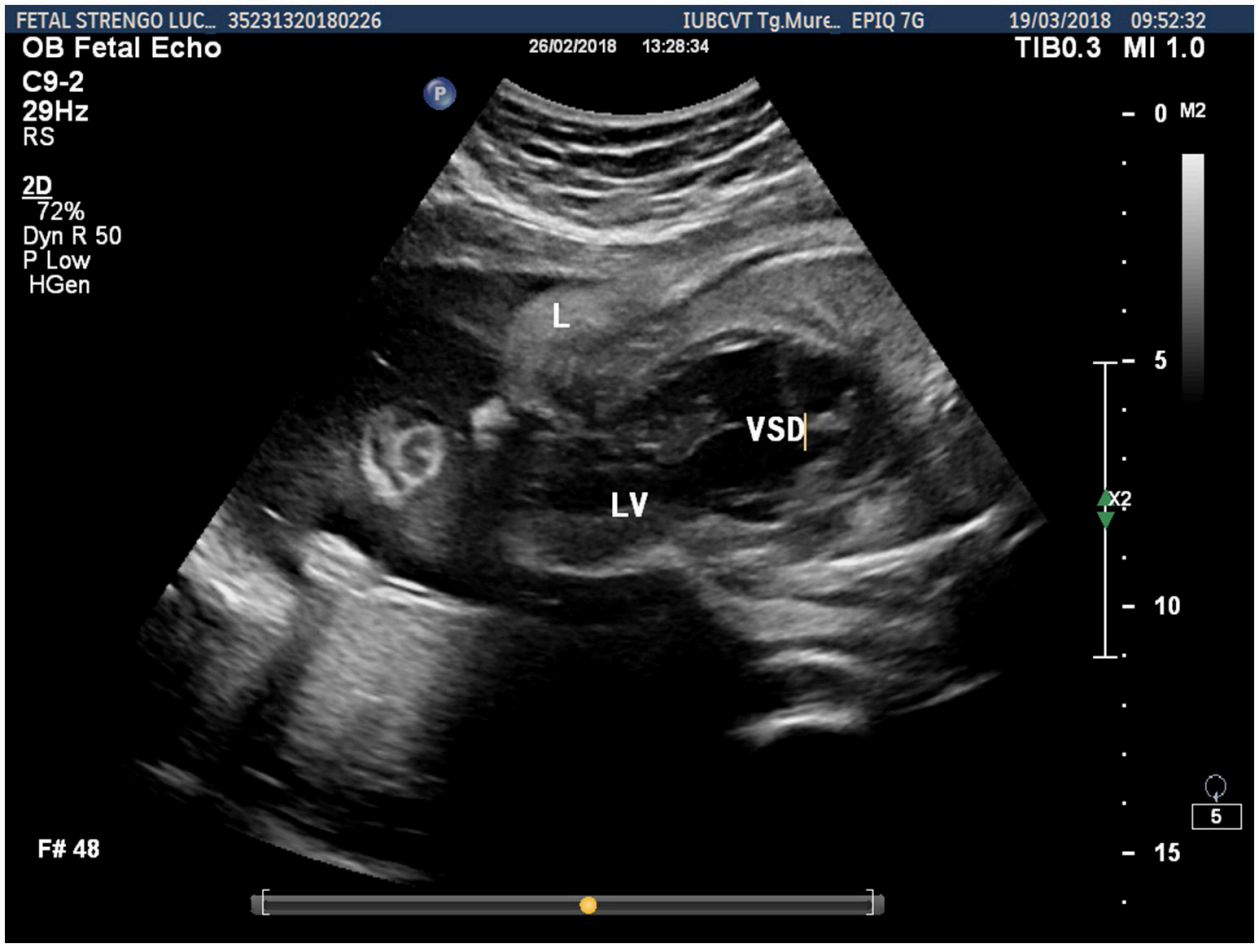
Fetal ultrasonographic image shows extrathoracic left ventricle and extra-abdominal liver (L, liver; LV, left ventricle; VSD, ventricular septal defect).

Prenatal ultrasonography revealed a fetal thoracoabdominal wall defect with partial displacement of the left ventricle and the liver associated with rotation and elongation of the heart and a high index of clinical suspicion for intracardiac malformations such as tricuspid atresia, a ventricular septal defect, and pulmonary artery hypoplasia (Figures 1–3).

### Clinical findings

**Figure 4:**
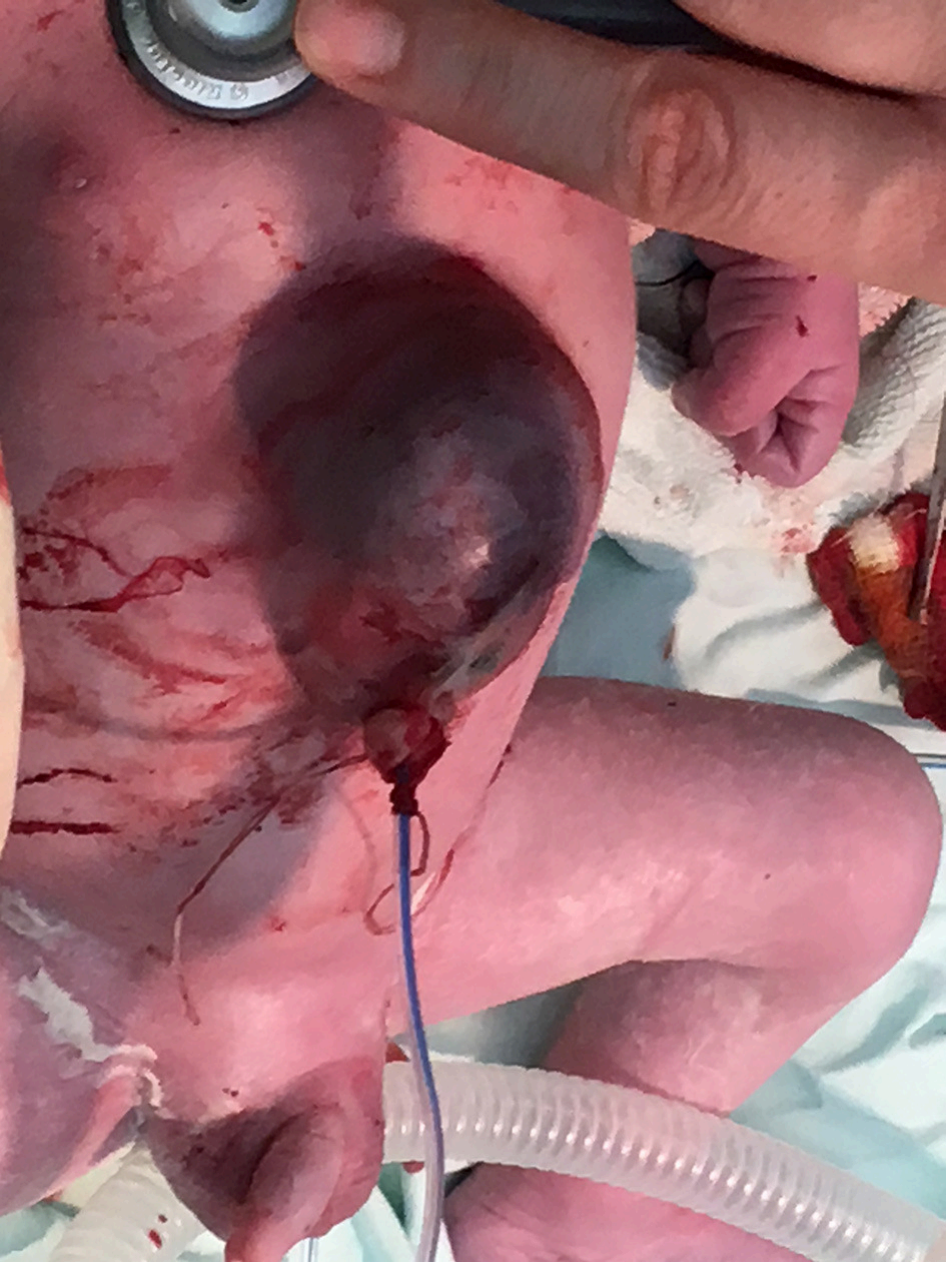
Clinical features of the newborn.

Based on the aforementioned findings, she was admitted to the Obstetrics and Gynecology Clinic in Târgu Mure at 39 gestational weeks, where she underwent a cesarean section. The male newborn weighed 3,100 g with an APGAR score of 7. Clinically, he demonstrated a superior abdominal wall defect, a partial extrathoracic displacement of the heart, and a partially herniated liver (these structures being covered by a very thin skin layer), and also a diastasis of the sagittal suture (Figure 4). The newborn was intubated, and we applied a saline-soaked gauze pad on the thoracoabdominal and cranial defects to maintain humidity.

### Diagnostic assessment

Postnatal echocardiography confirmed the prenatal diagnosis and also showed a partial extrathoracic and extra-abdominal displacement of the heart and liver, a large ventricular septal defect, great arteries originating from the left ventricle with the aorta situated anteriorly, a posterior deviation of the outlet septum causing severe subpulmonary stenosis, hypoplasia of the pulmonary artery, and a large hourglass-shaped left ventricle secondary to narrowing of the heart at the level of its extrathoracic displacement.

We also performed thoracoabdominal CT-angiography, which showed complex cardiac malformations consisting of large ventricular and atrial septal defects, an increased left ventricular volume, with apical extrathoracic aneurysmal dilatation below the xiphoid process at the level of the abdominal midline, hypoplasia of the right ventricle, and a reduced caliber of the pulmonary trunk artery. Abdominal CT revealed partial transparietal herniation of the left hepatic lobe adjacent to a left ventricular diverticulum, and an increase in the size of the right adrenal gland with hyperdense contents suggesting an adrenal hematoma. Cranial CT revealed a diastasis of the sagittal suture causing subcutaneous herniation of the venous sagittal sinus.

### Therapeutic intervention

Following admission to the Neonatal Intensive Care Unit, the newborn was administered ampicillin and amikacin, fluconazole, prostaglandin E, and phenobarbital (because he presented with multiple seizures), and also received daily dressing changes. During the first week of life, he showed multiple episodes of bradycardia and low oxygen saturation despite undergoing orotracheal intubation; therefore, surgical intervention was postponed until he was hemodynamically stable. He underwent surgical intervention at 14 days of age, consisting in the replacement of the heart inside the thorax via a systemico-pulmonary shunt procedure, with vascular prosthesis, the ligature of both persistent arterial canal and pulmonary artery trunk, and repair of the diaphragm defect. The abdominal wall defect was also sutured, but the thorax remained open. The surgical procedure was performed in extracorporeal circulation, and lasted 4 h and 15 min.

### Follow-up and outcome

Postoperatively, the newborn developed multiple episodes of tachyarrhythmia and low cardiac output suggesting an inability of the heart to adjust to the intrathoracic pressure. Unfortunately, the newborn died 5 h postoperatively secondary to progressive hemodynamic deterioration, metabolic acidosis, and hypoxia.

## Discussion

CS is an extremely rare condition. Prognosis depends upon the severity of the various complex components/anomalies that define this syndrome. The etiology of both EC and CS remains unknown, and most cases occur sporadically (12). The heart originally develops in the cephalic part of the embryo and reaches its final location by the 16th−17th day of embryogenesis, whereas complete developmental fusion leading to formation of the thoracic and abdominal cavities occurs by the 9th week of embryonic life (1, 13). Incomplete fusion can cause a wide range of disorders, varying from isolated EC to complete ventral evisceration (14). Different theories have been proposed to explain the etiology of this condition such as the amniotic rupture theory that describes EC with amniotic bands, although isolated EC may occur secondary to other etiological contributors such as exposure to intrauterine drugs (15). In our case, the mother denied any drug intake during pregnancy. Notably, a few cases of EC are reported to be associated with chromosomal abnormalities (16). In our case, there was no history of consanguinity, and genetic testing was negative.

Prenatal imaging studies are essential not only for prenatal counseling but also for adequate postnatal therapeutic planning. CS and EC can be easily diagnosed prenatally using two-dimensional (2D) or even three-dimensional (3D) ultrasonography combined with Doppler to ensure high accuracy. In a study performed on 12 patients with CS, the authors reported that 2D ultrasonography could establish the diagnosis in 11 patients, although 3D assessment provided additional information in all patients. Furthermore, they highlighted that the additional information can improve surgical management in these cases (17). Similarly, Desselle et al. suggested that the correlation between 2D and 3D examination is useful in surgical management in such newborns (2). The earliest prenatal diagnosis of CS is reported to have been established between 9 and 11 gestational weeks (18, 19). Unfortunately, in our case, the thoracic wall defect was suspected only at 35 gestational weeks. Therefore, we emphasize that accurate and timely prenatal diagnosis depends upon the sonographer's expertise in fetal ultrasonography. Pirasteh et al. also described a patient in whom intrauterine ultrasonography performed at 19 gestational weeks did not reveal any abnormalities, and the diagnosis of CS was established only after birth (20). Nevertheless, it is important to mention that although accurate prenatal diagnosis of this rare condition is essential for effective postnatal management, the timing of the prenatal diagnosis does not affect the outcome. In addition to ultrasonography, magnetic resonance imaging (MRI) is a useful diagnostic tool for prenatal diagnosis; however, it is useful only in selected cases. CT can be used when MRI is not available. We also performed CT, which confirmed the echocardiographic findings and provided additional information regarding intracardiac defects and revealed an apical aneurysmal dilatation of the extrathoracic heart. Abdominal CT additionally revealed a hematoma of the right adrenal gland.

EC can present with both, intracardiac and other associated anomalies. Intracardiac anomalies associated with EC, in the order of decreasing prevalence are ventricular septal defect, atrial septal defect, tetralogy of Fallot, a left ventricular diverticulum, and pulmonary hypoplasia (21). Our patient also presented with ventricular and atrial septal defects, pulmonary hypoplasia, and a left ventricular diverticulum, and additionally showed tricuspid atresia—an intracardiac defect that has not been reported previously in patients with EC described in the literature. In addition to the aforementioned intracardiac defects, other anomalies have been reported to be associated with EC such as trisomy 18, cleft lip and palate, neural tube defects, hydrocephaly, genitourinary malformations, pulmonary hypoplasia, abdominal wall defects varying from diastasis to omphalocele, as well as bowel and liver evisceration (22). Our patient also showed omphalocele and an ectopic liver, which concurs with studies reported in the literature; however, he additionally showed a diastasis of the sagittal suture causing herniation of the venous sagittal sinus. Türkçapar et al. described a case of a fetus with lumbosacral lordosis, a large omphalocele, a herniated liver, and EC (23).

The prognosis of CS and EC depends upon both, the complexity of the anomalies and the location of the ectopic heart. Based on the Toyama classification (8), we diagnosed our patient with a class II CS because the newborn presented with a midline supraumbilical abdominal wall defect and intracardiac anomalies without a pericardial or diaphragmatic defect. In contrast, a recent review of cases diagnosed with CS between 1998 and 2007 observed that class I CS was the most common type, accounting for >50% of the cases (24).

The surgical management of EC primarily involves 4 steps: soft tissue coverage of the heart, replacement of the heart into the thoracic cavity, repair of the intracardiac defects, and reconstruction of the chest wall (21). Therefore, optimal management of these patients requires coordination between a multidisciplinary team involving a neonatologist, a radiologist, a pediatric surgeon, a cardiologist, a pediatric cardiac surgeon, a plastic surgeon, and experienced nurses (25). Nevertheless, the general prognosis depends upon the promptitude of surgical intervention, which in our case was postponed owing to the patient's unstable status. Perhaps this fact led to the unfavorable prognosis in our patient, who underwent surgery only on the 14th day of life. Therefore, the patient died shortly after the procedure secondary to an inability of the heart to adjust to the intrathoracic conditions.

## Conclusions

Accurate prenatal diagnosis is essential for effective postnatal management of CS. In our case, the intrauterine ultrasonographic findings observed during the 3rd trimester were suggestive of CS. The complex cardiac malformations concomitant with other malformations worsened our patient's condition and prevented prompt surgical management during the early days of life.

## Table of contents summary

Cantrell syndrome is characterized by a midline anterior abdominal wall defect, defects in the diaphragm and in the apical pericardium, which communicates with the peritoneum, as well as intracardiac defects.

## Author contributions

CM, COM, and LM conceptualized and designed the study, drafted the initial manuscript, and reviewed and revised the manuscript. LG and MC designed the data collection instruments, collected data, carried out the initial analyses, reviewed and revised the manuscript. HS and HG, AC were involved in the surgical management, designed the data collection instruments, coordinated and supervised data collection, and critically reviewed the manuscript. All authors approved the final manuscript as submitted and agree to be accountable for all aspects of the work.

### Conflict of interest statement

The authors declare that the research was conducted in the absence of any commercial or financial relationships that could be construed as a potential conflict of interest. The reviewer EA and handling Editor declared their shared affiliation.

## Abbreviations

CS: Cantrell syndrome
EC: ectopia cordis
L: liver
LV: left ventricle
MV: mitral valve
PA: pulmonary artery
RV: right ventricle
TV: tricuspid valve
VSD: ventricular septal defect.

## References

[1] Cantrell JR Haller JA Ravitch MM. A syndrome of congenital defects involving the abdominal wall, sternum, diaphragm, pericardium, and heart. Surg Gynecol Obstet. (1958) 107:602–14. 13592660

[2] Desselle C Herve P Toutain A Lardy H Sembely C Perrotin F. Pentalogy of Cantrell: sonographic assessment. J Clin Ultrasound. (2007) 35:216–20. 10.1002/jcu.20318 17354250

[3] Luehr B Lipsett J Quinlivan JA. Limb-body wall complex: a case series. J Matern-Fetal Neonatal Med. (2002) 12:132–7. 10.1080/jmf.12.2.132.137 12420845

[4] Smigiel R Jakubiak A Lombardi MP Jaworski W Slezak R Patkowski D. Co-occurrence of severe Goltz-Gorlin syndrome and pentalogy of Cantrell–case report and review of the literature. Am J Med Genet A (2011) 155A:1102–5. 10.1002/ajmg.a.33895 21484999

[5] Maas SM Lombardi MP van Essen AJ Wakeling EL Castle B Temple IK. Phenotype and genotype in 17 patients with Goltz-Gorlin syndrome. J Med Genet. (2009) 46:716–20. 10.1136/jmg.2009.068403 19586929

[6] Barrott JJ Cash GM Smith AP Barrow JR Murtaugh LC. Deletion of mouse Porcn blocks Wnt ligand secretion and reveals an ectodermal etiology of human focal dermal hypoplasia/Goltz syndrome. Proc Natl Acad Sci USA. (2011) 108:12752–7. 10.1073/pnas.1006437108 21768372 PMC3150921

[7] Snowball J Ambalavanan M Cornett B Lang R Whitsett J Sinner D. Mesenchymal Wnt signaling promotes formation of sternum and thoracic body wall. Dev Biol. (2015) 401:264–75. 10.1016/j.ydbio.2015.02.014 25727890 PMC4424168

[8] Toyama WM. Combined congenital defects of the anterior abdominal wall, sternum, diaphragm, pericardium, and heart: a case report and review of the syndrome. Pediatrics (1972) 50:778–92. 4263752

[9] Pamidi N Vollala VR Nayak S Bhat S Case report-Ectopia cordis and amniotic band syndrome. Arch Med Sci. (2008) 4:208–11.

[10] Çelik Y Hallioglu O Basut N Demetgül H Esin Kibar A. A rare case of cardiac anomaly: prenatally diagnosed ectopia cordis. Turk Pediatri Arsivi. (2015) 50:129–31. 10.5152/tpa.2015.927 26265899 PMC4523987

[11] Zhang X Xing Q Sun J Hou X Kuang M Zhang G. Surgical treatment and outcomes of pentalogy of Cantrell in eight patients. J Pediatr Surg. (2014) 49:1335–40. 10.1016/j.jpedsurg.2014.06.003 25092102

[12] Goncalo FIMD Ana VRS Catia FCPM Ana PDF Joaquim MDF Ectopia cordis: caso clinic. Rev Bras Saude Materno Infant. (2014) 14:287–90. 10.1590/S1519-38292014000300010

[13] Shad J Budhwani K Biswas R. Thoracic ectopia cordis. BMJ Case Rep. (2012) 2012:1–4. 10.1136/bcr.11.2011.5241 23035158 PMC4543290

[14] Pius S Abubakar Ibrahim H Bello M Bashir Tahir M. Complete ectopia cordis: a case report and literature review. Case Rep Pediatr. (2017) 2017:1858621. 10.1155/2017/1858621 28503337 PMC5414485

[15] Jaffee OC Jaffee AL. Ectopia cordis in the chick embryo heart: an experimental study. Teratology (1990) 41:737–42. 10.1002/tera.1420410611 2353320

[16] Engum SA. Embryology, sternal clefts, ectopia cordis, and Cantrell's pentalogy. Semin Pediatr Surg. (2008) 17:154–60. 10.1053/j.sempedsurg.2008.03.004 18582820

[17] Bonilla-Musoles F Machado LE Bailão LA Osborne NG Raga F. Abdominal wall defects: two- versus three-dimensional ultrasonographic diagnosis. J Ultrasound Med Off J Am Inst Ultrasound Med. (2001) 20(4):379–89. 10.7863/jum.2001.20.4.379 11316317

[18] Bick D Markowitz RI Horwich A. Trisomy 18 associated with ectopia cordis and occipital meningocele. Am J Med Genet. (1988) 30:805–10. 10.1002/ajmg.1320300313 3189399

[19] Tongsong T Wanapirak C Sirivatanapa P Wongtrangan S. Prenatal sonographic diagnosis of ectopia cordis. J Clin Ultrasound (1999) 27:440–5. 10477886 10.1002/(sici)1097-0096(199910)27:8<440::aid-jcu5>3.0.co;2-6

[20] Pirasteh A Carcano C Kirsch J Mohammed T-LH. Pentalogy of Cantrell with ectopia cordis: CT findings. J Radiol Case Rep. (2014) 8:29–34. 10.3941/jrcr.v8i12.1972 25926914 PMC4394975

[21] Alphonso N Venugopal PS Deshpande R Anderson D. Complete thoracic ectopia cordis. Eur J Cardio-Thorac Surg. (2003) 23:426–8. 10.1016/s1010-7940(02)00811-4 12614821

[22] Taksande AM Vilhekar KY. A case report of ectopia cordis and omphalocele. Indian J Hum Genet. (2013) 19:491–3. 10.4103/0971-6866.124384 24497721 PMC3897151

[23] Türkçapar AF Sargin Oruc A Öksüzoglu A Danişman N. Diagnosis of pentalogy of cantrell in the first trimester using transvaginal sonography and color Doppler. Case Rep Obstet Gynecol. (2015) 2015:179298. 10.1155/2015/179298 25802780 PMC4352946

[24] Van Hoorn JHL Moonen RMJ Huysentruyt CJR van Heurn LWE Offermans JPM Mulder ALMT Pentalogy of Cantrell: two patients and a review to determine prognostic factors for optimal approach. Eur J Pediatr. (2008) 167:29–35. 10.1007/s00431-007-0578-9 17674044 PMC2668557

[25] Ramasauskaite D Snieckuviene V Zitkute V Vankeviciene R Lauzikiene D Drasutiene G. A rare case report of thoracic ectopia cordis: an obstetrician's point of view in multidisciplinary approach. Case Rep Pediatr. (2016) 2016:5097059. 10.1155/2016/5097059 27957373 PMC5120199

